# Supplementary material for: Splice-Junction-Based Mapping of Alternative Isoforms in the Human Proteome
Source: Cell Rep. Author manuscript; Available in PMC 2020 Jan 15. (PMC6961840; doi:10.1016/j.celrep.2019.11.026)
Supplement: 2 [file NIHMS1546469-supplement-2.zip › DF1/PXD009737/TestisTrypsin/TestisTrypsin_17_CMTR1_NSESHCSLQIK.pdf]

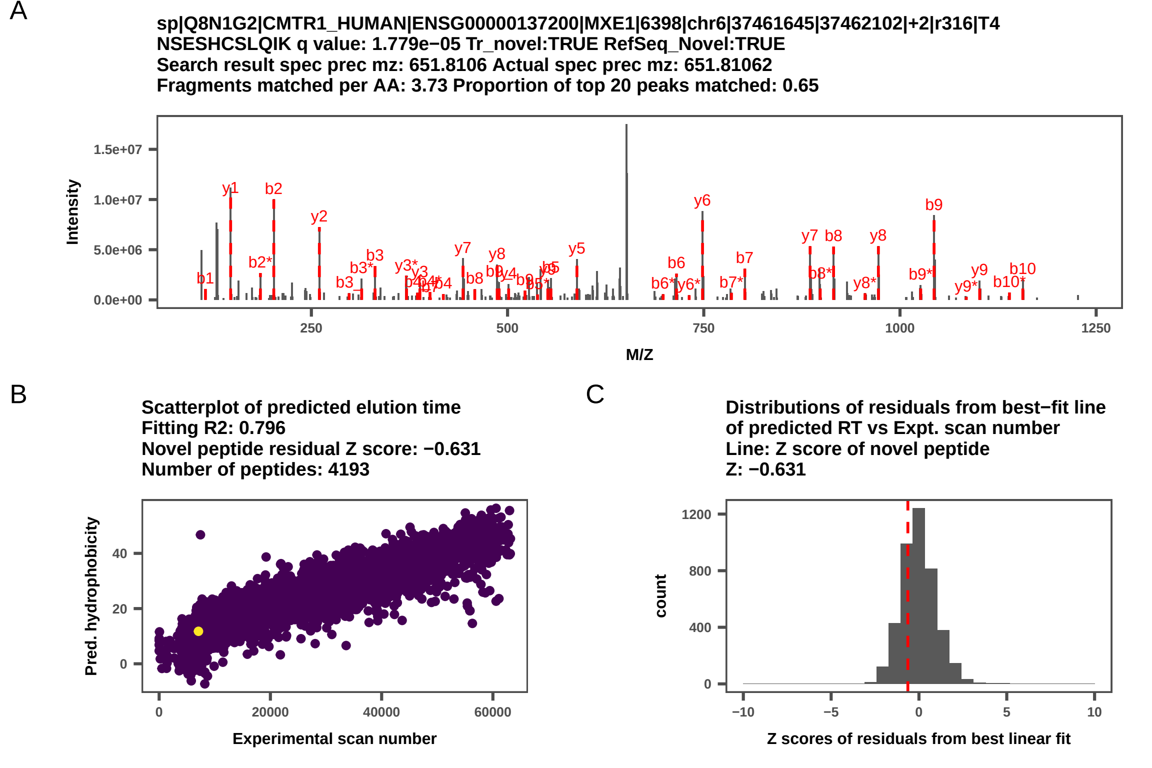
A
sp|Q8N1G2|CMTR1_HUMAN|ENSG00000137200|MXE1|6398|chr6|37461645|37462102|+2|r316|T4
NSESHCSLQIK q value: 1.779e−05 Tr_novel:TRUE RefSeq_Novel:TRUE
Search result spec prec mz: 651.8106 Actual spec prec mz: 651.81062
Fragments matched per AA: 3.73 Proportion of top 20 peaks matched: 0.65
Intensity
1.5e+07
1.0e+07
5.0e+06
0.0e+00
y1
b2
y2
b2*
b1
y6
b9
y7
b8
y8
y5
b6
b7
b3
b3*
y3*
y9
b10
b10*
y9*
b9*
y8*
b8*
b7*
b6*
y6*
250
500
750
1000
1250
M/Z
B
Scatterplot of predicted elution time
Fitting R2: 0.796
Novel peptide residual Z score: −0.631
Number of peptides: 4193
Pred. hydrophobicity
40
20
0
0
20000
40000
60000
Experimental scan number
C
Distributions of residuals from best−fit line
of predicted RT vs Expt. scan number
Line: Z score of novel peptide
Z: −0.631
count
1200
800
400
0
−10
−5
0
5
10
Z scores of residuals from best linear fit
